# DNA methyltransferase 3A promotes cell proliferation by silencing CDK inhibitor p18^INK4C^ in gastric carcinogenesis

**DOI:** 10.1038/srep13781

**Published:** 2015-09-09

**Authors:** He Cui, Chengcheng Zhao, Pihai Gong, Ling Wang, Huazhang Wu, Kun Zhang, Rongping Zhou, Li Wang, Ting Zhang, Sheng Zhong, Hong Fan

**Affiliations:** 1 Department of Medical Genetics and Developmental Biology, Medical School of Southeast University, The Key Laboratory of Developmental Genes and Human Diseases, Ministry of Education, Southeast University, Nanjing 210009, China; 2 The 3rd Affiliated Hospital of Harbin Medical University (Harbin Medical University Cancer Hospital), Harbin 150081, China; 3 The Affiliated Jiangning Hospital of Nanjing Medical University (Nanjing Jiangning Hospital), Nanjing 211100, China; 4 The Beijing Municipal Key Laboratory of Child Development and Nutriomics, Capital Institute of Pediatrics, Beijing 100020, China

## Abstract

Little is known about the roles of DNA methyltransferase 3A (DNMT3A) in gastric carcinogenesis. Here, we reported that the exogenous expression of DNMT3A promoted gastric cancer (GC) cell proliferation by accelerating the G1/S transition. Subsequently, p18^INK4C^ was identified as a downstream target of DNMT3A. The elevated expression of DNMT3A suppressed p18^INK4C^ at least at the transcriptional level. Depletion of p18^INK4C^ expression in GC cells induced cell cycle progression, whereas its re-expression alleviated the effect of DNMT3A overexpression on G1/S transition. Furthermore, we found that DNMT3A modulated p18^INK4C^ by directly binding to and silencing the p18^INK4C^ gene via promoter hypermethylation. In clinical GC tissue specimens analyzed, the level of methylation of p18^INK4C^ detected in tumor tissues was significantly higher than that in paired non-tumor tissues. Moreover, elevated level of DNMT3A expression was associated with the differentiation of GC tissues and was negatively correlated with the p18^INK4C^ expression level. Taken together, our results found that DNMT3A contributes to the dysregulation of the cell cycle by repressing p18^INK4C^ in a DNA methylation-dependent manner, suggesting that DNMT3A-p18^INK4C^ axis involved in GC. These findings provide new insights into gastric carcinogenesis and a potential therapeutic target for GC that may be further investigated in the future.

Carcinogenesis is a progression of events originating from the gradual accumulation of various genetic alterations and the disruption of epigenetic modifications^1^,^2^. DNA methylation is a major epigenetic mechanism that plays an important role in the early tumorigenic process^3^. Eukaryotic cells express three enzymatically active DNA methyltransferases (DNMTs), including DNMT1, DNMT3A and DNMT3B^4^. Previous studies have shown that DNMT1 and DNMT3B are intimately involved in the initiation and development of cancer^5^. However, the precise contribution of DNMT3A to tumorigenesis remains largely unknown. Gastric cancer (GC) is one of the most frequent malignancies in the world, especially in China, with a high incidence and mortality rate^6^,^7^. It has been reported that DNMT3A is ubiquitously overexpressed in multiple types of cancer, including GC^8^,^9^,^10^,^11^. Notably, the increased expression of DNMT3A in GC is significantly higher than that of DNMT1 and DNMT3B^9^,^12^. A recent study has demonstrated that the poor overall survival rate of GC patients is associated with elevated DNMT3A expression, but not with increased expression of DNMT1 or DNMT3B^13^. These findings indicate that the de-regulation of DNMT3A may be more critical for GC progression than that of the other two DNMTs. Many studies have shown that abnormal DNA methylation in GC alters the expression of tumor suppressor genes (TSGs)^14^,^15^,^16^,^17^. Therefore, further investigation of DNMT3A is needed to explore the precise role or mechanism underlying the regulation of GC.

In the gastrointestinal epithelium, cell proliferation and differentiation are highly regulated processes governed by intrinsic factors, such as cell cycle regulators^18^. Previous studies have demonstrated that inhibitors of CDK4 (INK4)-CDK4/6-CyclinD-Rb-E2F pathway play a key role in controlling cell growth^19^. The INK4 family includes p16^INK4A^, p15^INK4B^, p18^INK4C^, and p19^INK4D^, and its inactivation can lead to the formation of active CDK4/6-CyclinD complexes and further promote cell cycle progression^20^. In GC, the de-regulation of p16 has been shown to significantly increase the risk of malignant transformation of gastric epithelial cells^21^, and the silencing of INK4 members induced by Ras homolog family member A (RhoA) has been associated with G1/S progression, indicating that INK4 members are involved in GC cell proliferation^22^. In addition, the silencing of INK4 members via promoter hypermethylation has been shown to occur in certain cancers^23^,^24^,^25^. However, it remains unclear whether the increased expression of DNMT3A in GC accounts for the dysregulation of INK4 members.

In this study, we investigated the expression pattern and biological function of DNMT3A in GC as well as DNA methylation mechanism resulting from its activity. We have shown that DNMT3A is involved in GC progression via methylation of the p18^INK4C^ promoter, which leads to the downregulation of p18^INK4C^, thereby disrupting the G1/S checkpoint and eventually promoting GC cell proliferation. These findings may be beneficial to the development of new treatment options for GC that target DNMT3A.

## Results

### DNMT3A is important for GC cell proliferation

**Figure 1:**
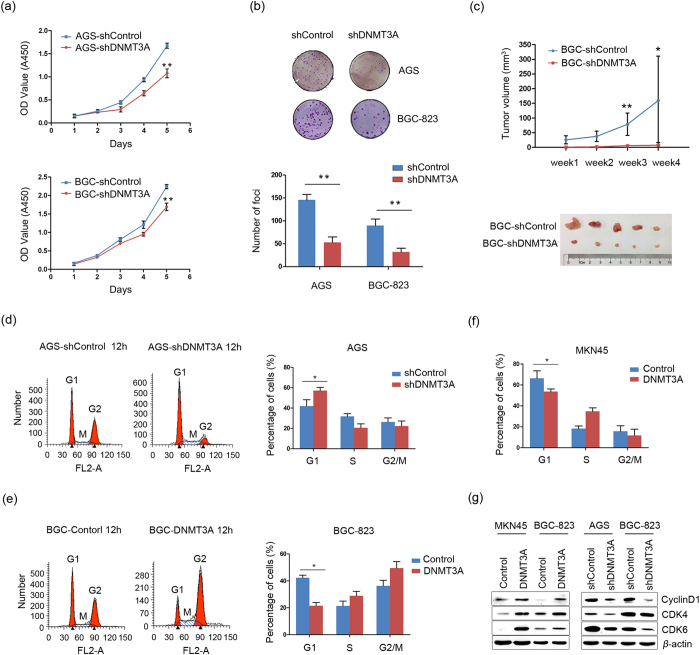
DNMT3A has tumor-promoting effects *in vitro* and *in vivo*. (**a**) The growth rates of AGS-shDNMT3A and BGC-shDNMT3A cells were detected with CCK-8 proliferation assay and are shown as the mean ± SD of three independent experiments (**P < 0.01). (**b**) Representative images of foci formation in AGS-shDNMT3A and BGC-shDNMT3A cells (upper panels). Colonies were counted, and the results are depicted in a bar chart (bottom panels). The values indicate the mean ± SD of three independent experiments (**P < 0.01). (**c**) BGC-shControl or BGC-shDNMT3A cells were injected into the right dorsal flanks of nude mice (n = 5 per group), respectively. The tumor growth curve shows that DNMT3A knockdown significantly inhibited tumor growth in the mice (upper panels; *P < 0.05, **P < 0.01). Images of tumors formed in the nude mice injected with the indicated cells are also shown (bottom panels). (**d**) Flow cytometry analysis of the distribution of cell cycle phases in AGS-shControl and AGS-shDNMT3A cells (left panels). The percentages of cells in the G1, S, and G2/M phases are shown in the bar chart as the mean ± SD of three independent experiments (right panels; *P < 0.05). (**e**) Flow cytometry analysis of the distribution of cell cycle phases in BGC-Control and BGC-DNMT3A cells (left panels). The percentages of cells in the G1, S, and G2/M phases are shown in the bar chart as the mean ± SD of three independent experiments (right panels; *P < 0.05). (**f**) The percentages of MKN45-Control and MKN45-DNMT3A cells in the G1, S, and G2/M phases are shown in the bar chart as the mean ± SD of three independent experiments (*P < 0.05). (**g**) Expression levels of cyclinD1, CDK4 and CDK6 were detected in DNMT3A overexpression MKN45 and BGC-823 cells (left panels) or in DNMT3A knockdown AGS and BGC-823 cells by western blot assay (right panels). *β*-actin was used as a loading control.

Abnormal cell proliferation is a characteristic feature of cells that have undergone malignant transformation. DNMT3A has been implicated in cell survival in melanoma and hepatocellular carcinoma^26^,^27^. To evaluate the functional outcomes of DNMT3A in GC progression, a cell model for DNMT3A analysis was generated. AGS and BGC-823 cells were selected to establish stable DNMT3A knockdown GC cell lines (named AGS-shDNTM3A and BGC-shDNTM3A). Compared with control cells (named AGS-shControl and BGC-shControl), DNMT3A protein expression was dramatically decreased in AGS-shDNTM3A and BGC-shDNTM3A cells (Figure S1a). The biological roles of DNMT3A were then assessed via cell growth rate and foci formation assays *in vitro*. The results showed that DNMT3A knockdown AGS and BGC-823 cells had significantly reduced growth rates (*P *< 0.01; Fig. 1a) and lower frequencies of foci formation (*P *< 0.01; Fig. 1b) compared with control cells. Conversely, we examined the effect of the ectopic expression of DNMT3A on the growth of MKN45 cells. Exogenous DNMT3A expression in stably transfected MKN45 cells (named MKN45-DNMT3A) was confirmed by western blot analysis (Figure S1b). DNMT3A overexpression caused a significant increase in the cell growth rate (*P *< 0.05; Figure S2a), suggesting that DNMT3A is required for GC cell proliferation. To further investigate the contribution of DNMT3A to cell proliferation, we investigated its role in nude mice *in vivo*. The tumor growth curves of BGC-shControl and BGC-shDNMT3A cells in nude mice are shown in Fig. 1c. The tumor volume was significantly smaller in BGC-shDNMT3A cell-injected nude mice (7.5 ± 9.23 mm^3^) than in BGC-shControl cell-injected mice (159.5 ± 151.26 mm^3^; *P *< 0.05; Fig. 1c and Figure S2b), indicating that DNMT3A has a critical function in tumor growth. Collectively, these results demonstrate that DNMT3A promotes tumor cell proliferation *in vitro* and *in vivo* and therefore may contribute to maintaining malignant phenotype in GC.

### DNMT3A promotes cell growth by disrupting the G1/S checkpoint

De-regulation of cell growth in tumor cells is usually associated with the concomitant acceleration of cell cycle progression. We therefore examined the contribution of the cell cycle to the observed growth rate induced by DNMT3A. AGS-shControl and AGS-shDNMT3A cells were synchronized, with most cells in the G1 phase (72%). After being released from synchronized for 12 h, the percentage of cells in the G1 phase was significantly higher for AGS-shDNMT3A cells than for AGS-shControl cells (57.16% versus 41.93%; *P *< 0.05; Fig. 1d). Reciprocally, we synchronized BGC-823 cells stably transfected with a DNMT3A construct (named BGC-DNMT3A cells) or empty vector (named BGC-Control cells) (Figure S1b), with most cells in the G1 phase (70%). After being released from synchronized for 12 h, the percentage of cells in the G1 phase was significantly lower for BGC-DNMT3A cells than for BGC-Control cells (21.52% versus 42.30%; *P *< 0.05; Fig. 1e). This effect was also observed in untreated MKN45-DNMT3A cells, which showed a significantly decreased percentage of cells in the G1 phase compared with MKN45-Control cells (53.60% versus 66.21%; *P *< 0.05; Fig. 1f). To elucidate the molecular basis of the acceleration of the G1/S transition, we examined several G1/S regulators in DNMT3A overexpression cells or knockdown cells. The results showed that the levels of cyclinD1, CDK4 and CDK6 were increased in DNMT3A-induced MKN45 and BGC-823 cells. Conversely, inhibition of DNMT3A reduced the protein levels of cyclinD1, CDK4 and CDK6 (Fig. 1g), suggesting that DNMT3A stimulated cell cycle progression by disrupting the G1/S checkpoint.

### Identification of target genes modulated by DNMT3A

**Figure 2:**
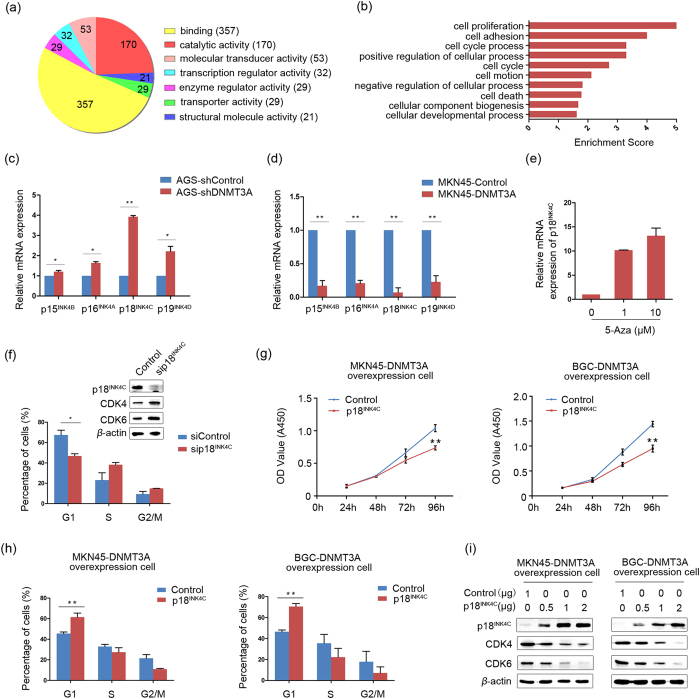
p18^INK4C^ participates in DNMT3A-induced cell proliferation. (**a**) The pie chart shows the top seven most significantly enriched molecular function gene ontology (GO) terms. (**b**) The bar plot shows the top ten most significantly enriched biological process GO terms and their scores. Count, the number of differentially expressed genes associated with the listed GO ID. Enrichment Score, the GO ID enrichment score, which equals (−log_10_ (*P*-value)). (**c**,**d**) Relative mRNA expression of INK4 members was detected in DNMT3A knockdown AGS cells or in DNMT3A overexpression MKN45 cells by qPCR*. β*-actin was used as an internal control (*P < 0.05, **P < 0.01). (**e**) The mRNA expression of p18^INK4C^ was detected in DNMT3A overexpression MKN45 cells after treatments with different doses of 5-Aza. (**f**) Flow cytometry analysis of the distribution of cell cycle phases after treatment of GC cells with sip18^INK4C^. The percentages of cells in the G1, S, and G2/M phases are shown in the bar chart as the mean ± SD of three independent experiments (*P < 0.05). After the silencing of p18^INK4C^ by treatment with sip18^INK4C^, the protein expression levels of CDK4 and CDK6 were increased. *β*-actin was used as a loading control. (**g**) CCK-8 analysis of cell proliferation in DNMT3A overexpression MKN45 and BGC-823 cells transiently transfected with a p18^INK4C^ construct and control vector. The cell growth rates are shown as the mean ± SD of three independent experiments (**P < 0.01). (**h**) Flow cytometry analysis of cell cycle phases in DNMT3A overexpression MKN45 and BGC-823 cells transiently transfected with a p18^INK4C^ construct and control vector. The percentages of cells in the G1, S, and G2/M phases are shown as the mean ± SD of three independent experiments (**P < 0.01). (**i**) Transient transfection of p18^INK4C^ into DNMT3A overexpression MKN45 and BGC-823 cells led to dose-dependent downregulation of CDK4 and CDK6, as determined by western blotting. *β*-actin was used as a loading control.

The finding of a critical function of DNMT3A in the cell cycle motivated us to identify its target genes associated with the G1/S transition. Differential expression profiles between control and MKN45-DNMT3A cells were examined via complementary DNA (cDNA) microarray. Results showed that 906 genes expression level were remarkably altered (fold-change cutoff: 2.0). Of these altered genes, 558 genes were downregulated and 348 genes were upregulated. Gene ontology (GO) analysis of the dysregulated genes indicated that the predominant molecular functions were binding and catalytic activity (Fig. 2a). In the biological processes category, the top ten largest groups of dysregulated genes in response to DNMT3A overexpression were assigned to the GO terms cell proliferation, cell adhesion, cell cycle process and other processes (Fig. 2b), indicating that DNMT3A plays an oncogenic role in these processes.

According to the above analysis, 5 downregulated genes related to cell cycle were found, including E2F2, RBL1, RHOU, PRIM1 and BLM. To better gain insights into the molecular mechanism of the G1/S transition by DNMT3A, we added 22 downregulated genes as candidate by adjusting fold-change cutoff of the microarray. GO analysis revealed that 5 of 27 genes, including BLM, CDKN1B, CCNE2, p18^INK4C^ and CKS1B, were associated with the regulation of CDK activity. Of the 5 genes fulfilling this criterion, CDKN1B and p18^INK4C^ have been previously implicated in processes relevant to the inhibition of cyclinD1 and CDK4/6 complex formation during the G1 phase^20^. However, knockdown of DNMT3A expression did not have a remarkable effect on the expression of CDKN1B at the protein levels (Figure S3a), suggesting that p18^INK4C^ may be a target of DNMT3A-mediated dysregulation of cell cycle. To further validate the microarray results for p18^INK4C^, quantitative real-time PCR (qPCR) was used to investigate the effect of DNMT3A on p18^INK4C^ expression, along with the other INK4 members, including p15^INK4B^, p16^INK4A^ and p19^INK4D^. The results showed that the depletion of DNMT3A increased the transcriptional levels of the four members of the INK4 family (Fig. 2c). Conversely, the suppression of the INK4 members was observed in DNMT3A-induced GC cells (Fig. 2d). Notably, p18^INK4C^ expression was more significantly changed than that of the other INK4 members. We further found that the effect of DNMT3A on p18^INK4C^ inhibition could be reversed by treatment of MKN45-DNMT3A cells with the DNA methyltransferase inhibitor 5-azacytidine (5-Aza) (Fig. 2e), suggesting that the p18^INK4C^ gene is the major downstream target of DNMT3A.

DNMT1 and DNMT3B have been associated with TSGs silencing in human tumors. To confirm that the suppression of p18^INK4C^ is specific to DNMT3A overexpression, the mRNA levels of INK4 members were detected following the silencing of DNMT3B or DNMT1 in the AGS cell line by RNAi using siRNA against DNMT3B or DNMT1. The results showed that the expression of p19^INK4D^ was increased in DNMT3B knockdown cells (defined as a greater than 2-fold increase), whereas no significant changes were observed in the mRNA levels of the other INK4 members (Figure S4a). In addition, the depletion of DNMT1 increased the transcriptional levels of four members of the INK4 family (Figure S4b). However, the increases in the expression levels of p16^INK4A^, p15^INK4B^ and p19^INK4D^ were higher than that of p18^INK4C^ in DNMT1 knockdown cells. Although the expression of p18^INK4C^ was partially affected by DNMT1, no significant upregulation of DNMT1 (*P *> 0.05, Figure S4c) or DNMT3B (*P *> 0.05, Figure S4d) mRNA levels was observed in 35 GC tissues (the same tissues used to detect DNMT3A expression) compared with non-tumor tissues. Likewise, we did not find significant increases in DNMT3B or DNMT1 expression in manipulated AGS, BGC-823 or MKN45 cells used in this study (*P *> 0.05, Figure S4e). Collectively, these data indicate that the suppressive effect of p18^INK4C^ is due to DNMT3A and not to DNMT1 or DNMT3B.

### p18^INK4C^ is involved in DNMT3A-mediated G1/S transition

To confirm that the DNMT3A-p18^INK4C^ axis contributes to cell cycle regulation, we tested whether p18^INK4C^ arrests GC cells at the G1/S phase. Flow cytometry analysis revealed that depletion of p18^INK4C^ with a specific siRNA (named sip18^INK4C^ cells) increased the G1/S transition compared with a negative control siRNA (named siControl cells) (*P *< 0.05; Fig. 2f). Western blot analysis showed significant increases in the protein levels of CDK4 and CDK6 in p18^INK4C^ knockdown cells (Fig. 2f). Subsequently, we investigated whether enforced p18^INK4C^ expression in DNMT3A overexpression cells could reduce cell growth. Compared with control cells, both MKN45-DNMT3A and BGC-DNMT3A cells showed significantly reduced cell growth rates (*P *< 0.01; Fig. 2g) and restoration of the percentages of cells in the G1 phase (*P *< 0.01; Fig. 2h) after re-expression of p18^INK4C^. Moreover, the protein levels of CDK4 and CDK6 were found to be repressed in dose-dependent manners by the ectopic expression of p18^INK4C^ (Fig. 2i), suggesting that the restoration of p18^INK4C^ expression effectively prevented CDK4 and CDK6 activation and delayed the cell cycle progression mediated by DNMT3A overexpression. Collectively, these data indicated that p18^INK4C^ participated in the DNMT3A-induced G1/S transition.

### Repression of p18^INK4C^ via DNMT3A-mediated DNA methylation

**Figure 3:**
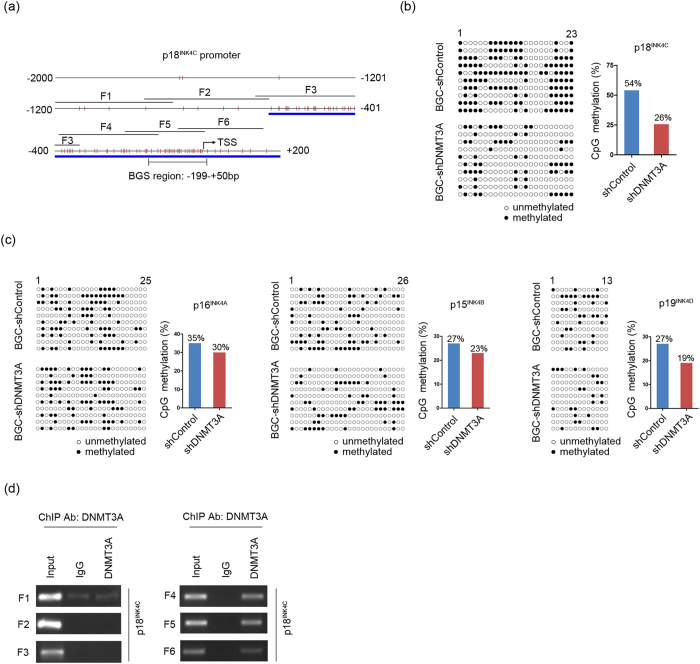
DNMT3A suppresses p18^INK4C^ in a DNA methylation-dependent manner. (**a**) Diagram of the promoter of the p18^INK4C^ gene, with the transcription start site (TSS) indicated. The blue line indicates the CpG island, and the red vertical line depicts a CpG site. One region (−199 bp ~ +50 bp) spanning a CpG island with 23 CpG sites was analyzed. The black line represents the location of the F1~F6 fragment detected by ChIP assay. (**b,c**) Methylation statuses of the INK4 member promoters in BGC-shControl and BGC-shDNMT3A cells, as detected by BGS assay (left panels). ○ represents unmethylated CpG sites; and ● represents methylated CpG sites. Each row represents a single sequence. The bar graphs depict the INK4 member promoter methylation rates (right panels). (**d**) ChIP assay was performed using a DNMT3A-specific antibody, followed by PCR amplification of six individual fragments representing p18^INK4C^ promoter regions (F1–F6). Chromatin (defined as Input) and products immunoprecipitated by IgG were used as positive and negative controls, respectively.

To evaluate whether DNA methylation is involved in DNMT3A-induced p18^INK4C^ silencing, bisulfite DNA sequencing (BGS) assay was performed to examine the methylation status of the promoter region of p18^INK4C^ in DNMT3A-depleted BGC cells. As shown in Fig. 3a, 23 individual CpG sites within CpG island regions (from −199 to +50 bp) were sequenced to identify methylated cytosine residues. The results showed that the frequency of p18^INK4C^ promoter methylation in BGC-shDNMT3A cells was 26%, which was significantly lower than the level measured in control cells (54%; defined as a greater than 2-fold decrease) (Fig. 3b). Furthermore, BGS analysis was performed to evaluate the other three INK4 members (Fig. 3c).The promoters of these members did not show marked decreases in the numbers of methylated CpG sites, indicating that DNMT3A-mediated promoter hypermethylation mainly inactivates p18^INK4C^ among the INK4 members. To detect a possible physical interaction between DNMT3A and the promoter region of p18^INK4C^, ChIP assay was performed using antibodies against DNMT3A or control IgG. As shown in Fig. 3a, we designed a panel of six primer pairs that covered a 1.5-kb region of the p18^INK4C^ promoter (−1200/+201; F1: −1191/−892; F2: −967/−643; F3: −683/−333; F4: −406/−148; F5: −259/+18 and F6: −90/+151) to probe fragments via PCR. The F4, F5, and F6 fragments were detected and found to be abundantly bound to DNMT3A in DNMT3A-ChIPed DNA (Fig. 3d). Specifically, the DNMT3A-binding regions (−406/+151) overlapped with the CpG island of the p18^INK4C^ promoter, indicating that the direct binding of DNMT3A regulates p18^INK4C^ expression in a methylation-dependent manner.

### Abnormal p18^INK4C^ methylation in clinical GC specimens

**Figure 4:**
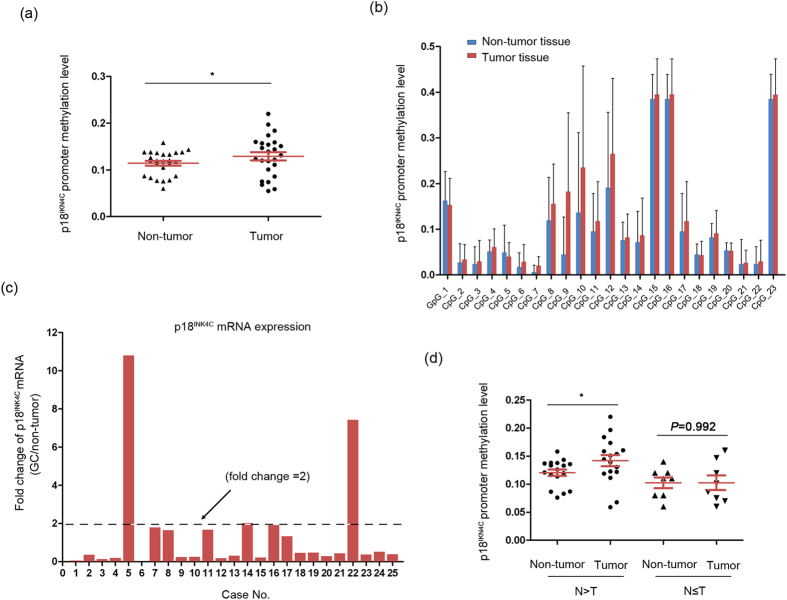
The methylation status of p18^INK4C^ promoter in clinical GC specimens. (**a**) Scatter plots of p18^INK4C^ promoter methylation levels in 25 paired GC tissues (T) and adjacent non-tumor tissues (N) (*P < 0.05). In both panels, the red lines indicate the mean ± SEM. (**b**) The methylation levels of 23 CpG sites in 25 paired GC tissues and adjacent non-tumor tissues. (**c**) qPCR analysis showing p18^INK4C^ mRNA levels in 25 paired clinical GC specimens. The value (defined as “fold change”) indicates the ratio of the p18^INK4C^ mRNA expression levels in the GC versus non-tumor tissues. (**d**) A total of 25 paired clinical GC specimens were divided into two subgroups based on their relative p18^INK4C^ mRNA expression levels. Tissues with low p18^INK4C^ expression (defined as a less than 2-fold decrease) were assigned to the N > T group, while the remainder were classified into the N ≤ T group. The tissues with low p18^INK4C^ expression display higher methylation levels in tumor tissues compared with adjacent non-tumor tissues (*P < 0.05).

To further determine whether the abnormal methylation of p18^INK4C^ is involved in clinical GC progression, we investigated the methylation statuses of 23 individual CpG sites within p18^INK4C^ promoter regions in 25 pairs of GC cases and adjacent non-tumor controls. We found that the average methylation level of p18^INK4C^ was significantly elevated in tumor tissues compared with non-tumor tissues (*P *< 0.05; Fig. 4a). Moreover, although each site displayed extensive methylation in tumor and adjacent non-tumor tissues, the methylation levels of 19 sites within the p18^INK4C^ promoter CpG island were higher in tumor tissues than in non-tumor tissues, especially CpG 7 and 9 (defined as a greater than 2-fold increase; Fig. 4b). Furthermore, we detected the mRNA expression of p18^INK4C^ in the above cases (Fig. 4c). Then, the GC patients were divided into two subgroups based on their relative p18^INK4C^ mRNA status. Interestingly, in the clinical cases with low p18^INK4C^ expression (defined as a less than 2-fold decrease in GC tissues compared with adjacent non-tumor tissues), the p18^INK4C^ promoter showed significantly higher methylation levels in tumor tissues than in adjacent non-tumor tissues, indicating a negative correlation between DNA methylation and p18^INK4C^ expression (*P *< 0.05; Fig. 4d). Thus, the methylation-associated silencing of p18^INK4C^ is involved in GC progression, suggesting that the DNMT3A-p18^INK4C^ axis may be an important regulator of gastric carcinogenesis.

### Correlation of DNMT3A with p18^INK4C^ expression in clinical GC specimens

**Figure 5:**
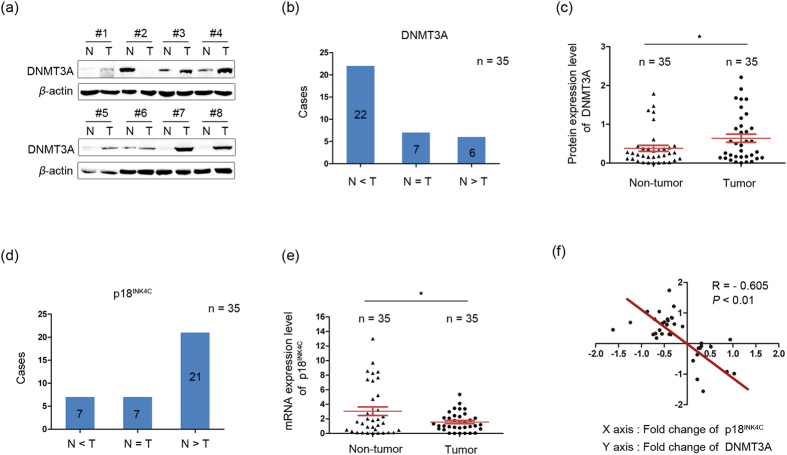
Elevated DNMT3A expression is correlated with p18^INK4C^ downregulation in clinical GC specimens. (**a**) Western blot analysis of DNMT3A in 8 representative GC tissues (T) and paired adjacent non-tumor (N) tissues. *β*-actin was used as a loading control. (**b**) The relative protein expression levels of DNMT3A in 35 paired GC and adjacent non-tumor tissues divided into three groups (N > T, N = T and N < T) based on relative expression scores of greater than or less than 2-fold. The number of cases is shown for every group. (**c**) Scatter plots of relative DNMT3A protein expression in 35 GC and paired adjacent non-tumor tissues. In both panels, the red lines indicate the mean ± SEM (*P < 0.05). All protein samples were derived from the same experiment, and SDS-PAGE gels were processed in parallel. (**d**) The relative mRNA expression levels of p18^INK4C^ in 35 paired GC and adjacent non-tumor tissues divided into three groups (N > T, N = T and N < T) based on relative expression scores of greater than or less than 2-fold. The number of cases is shown for each group. (**e**) Scatter plots of relative p18^INK4C^ mRNA expression in 35 GC and adjacent non-tumor tissues. In both panels, the red lines indicate the mean ± SEM (*P < 0.05). (**f**) Correlation between DNMT3A protein and p18^INK4C^ mRNA expression in 35 clinical samples, with linear regression lines and correlation significance (R = −0.605; **P < 0.01).

The finding of a critical function of DNMT3A in GC cells implicates the involvement of DNMT3A in GC progression. DNMT3A expression was detected in 35 pairs of GC cases and quantified by measuring band intensities with *ImageJ* software. Compared with the paired adjacent non-tumor tissues, DNMT3A was overexpressed (defined as a greater than a 2-fold increase) in 22 of the 35 (63%) samples, according to the western blot data (Fig. 5a,b). The average expression level of DNMT3A in the tumor tissues was significantly higher than that in the paired adjacent non-tumor tissues (0.68 versus 0.39; *P *< 0.05; Fig. 5c). The correlation between DNMT3A protein expression and GC clinical pathological features was then analyzed. A total of 35 patients were categorized into two groups according to the DNMT3A protein expression level. The patients with DNMT3A overexpression (defined as a greater than 2-fold increase) were assigned to the N <T group, while the remainder were classified into the N ≥T group. The results revealed that DNMT3A overexpression was strongly correlated with tumor cell differentiation (Table 1). These data indicate that the elevated expression of DNMT3A is important for gastric carcinogenesis. Furthermore, we explored the relationship between DNMT3A protein expression and p18^INK4C^ mRNA expression. A total of 35 pairs of GC cases and adjacent non-tumor controls were used for the detection of p18^INK4C^ by qPCR. The results showed that the frequency of p18^INK4C^ downregulation (defined as a greater than 2-fold decrease) was 60% (21/35; Fig. 5d) and that its average expression in tumor tissues was significantly lower than that in paired adjacent non-tumor tissues (1.56 versus 3.05; *P *< 0.05; Fig. 5e). In addition, an association study showed that p18^INK4C^ mRNA expression was negatively correlated with DNMT3A protein expression in the 35 pairs of GC cases (*R *= −0.605; *P *< 0.01; Fig. 5f).

## Discussion

GC is one of the most frequently occurring, fatal malignancies worldwide (ranking 4^th^ for males and 5^th^ for females), and the prognosis of this disease remains very poor^6^. Thus, a better understanding of the molecular mechanisms involved in GC progression is urgently required. Gene silencing via the hypermethylation of promoter CpG islands is a common event in various human cancers^28^. Previous studies have shown that the suppression of DNMT3A dramatically inhibits melanoma growth via de-regulation of cell cycle-related genes in mouse melanoma^26^. Similarly, DNMT3A-mediated promoter methylation causes *PTEN* gene silencing and promotes hepatocellular carcinoma cell proliferation^27^. In addition, elevated DNMT3a activity promoted polyposis in *Apc*^*Min*^ mice^29^. All of these results indicate that DNMT3A facilitates tumor development. In GC, DNMT3A is enriched in tumor tissues, and it is recognized as an independent poor prognostic factor^12^,^13^; thus, it is important to assess its biological function and the molecular mechanism underlying its promotion of GC individually. In this study, cell growth curve, colony-forming and *in vivo* tumorigenicity assays were employed to assess the characteristics of GC cells induced by DNMT3A. The results revealed that DNMT3A suppression in GC cell lines decreased cell proliferation, inhibited colony formation and suppressed tumorigenicity in nude mice. Furthermore, we found that the effects of DNMT3A overexpression were related to dysregulation of the G1/S transition by upregulation of CDK4 and CDK6 expression. DNMT3A was found to have tumor-promoting effects both *in vitro* and *in vivo*, and these results were concordant with those of our subsequent GO analysis of gene expression profiles based on microarray data, which revealed the top ten most significantly enriched categories, including cell proliferation, cell adhesion, cell cycle process and other processes.

Previous studies have demonstrated that several target genes, such as *PTEN* and insulin-like growth factor-3, are regulated by DNMT3A in hepatocellular carcinoma^27^,^30^. However, the target specificity of DNMT3A in GC remains unclear. In this study, we found that DNMT3A altered the expression of the cell cycle-related molecule p18^INK4C^ by microarray analysis. Further validation revealed that the increased expression of p18^INK4C^ in DNMT3A-depleted GC cells was significantly higher than that of three other INK4 members, implying that p18^INK4C^ may be a major downstream gene involved in the DNMT3A-induced G1/S transition. The most critical point in cell cycle regulation is the G1/S checkpoint, which is controlled by cyclins, CDKs, and CDK-inhibitors (CKIs)^31^. Members of the INK4 family of CKIs function as negative regulators of kinase activities^32^. p18^INK4C^, a member of the INK4 family, functions as a tumor suppressor in mice, and its deletion has been associated with the development of tumors when combined with other oncogenic stimuli^33^,^34^,^35^. Similarly, the finding of decreased p18^INK4C^ gene expression at high frequencies in human cancers further supports its negative regulatory role during tumorigenesis^36^,^37^,^38^. Here, we found that p18^INK4C^ was required for the G1/S transition and that its re-expression reduced cell growth and delayed cell cycle progression from the G1 to S phase by inactivating CDK4 and CDK6 in DNMT3A-overexpressing cells, indicating that a functional connection exists between p18^INK4C^ and DNMT3A.

All INK4 members contain a CpG island in their promoter, suggesting that DNA methylation may contribute to the silencing of these genes. Previous studies have shown that a point mutation or deletion rarely inactivates the p18^INK4C^ locus^39^, suggesting that one of the mechanisms of p18^INK4C^ inactivation is epigenetic silencing. Indeed, promoter hypermethylation of p18^INK4C^ has been reported in certain cancers^23^,^24^,^25^. Thus, we assessed the methylation statuses of INK4 members. Interestingly, the methylation level of p18^INK4C^ was more readily restored than those of the other three INK4 members due to promoter demethylation via DNMT3A suppression, indicating that the silencing of p18^INK4C^ is mainly mediated by DNMT3A-dependent methylation. However, the levels of p16^INK4A^, p15^INK4B^ and p19^INK4D^ demethylation were not remarkably increased in DNMT3A-depleted cells, suggesting that some unknown mechanism, such as histone modification combined with DNA methylation, may be involved in the regulation of these genes^40^,^41^,^42^,^43^,^44^. To further confirm the relationship between DNMT3A and p18^INK4C^, a subsequent ChIP assay demonstrated a striking preference of DNMT3A for p18^INK4C^. Thus, DNMT3A functions by binding to and catalyzing the methylation of the p18^INK4C^ promoter in GC cells. However, the precise processes need to be explored in further studies. In this aspect, putative binding sites of Sp1 in the promoter of the p18^INK4C^ gene have been reported to be critical for p18^INK4C^ expression^45^. In this study, we did not find any significant changes in the predicted Sp1 binding sites in DNMT3A-depleted cells or control cells, indicating that aberrant methylation caused p18^INK4C^ silencing by preventing the binding of other transcription factors.

We next examined the expression pattern and methylation status of p18^INK4C^ in clinical GC tissue specimens. The percentage of methylated CpGs located within p18^INK4C^ was higher in tumor tissues than in paired adjacent non-tumor tissues. These data are consistent with our findings in GC cells, indicating that DNA methylation is a key regulatory mechanism of p18^INK4C^ silencing. In addition, we assessed DNMT3A expression by western blot assay, which differs from the previous detection method using immunohistochemistry. The results showed that DNMT3A overexpression was significantly positively correlated with cell differentiation in GC and that it was negatively correlated with decreased p18^INK4C^ levels. In summary, our study has revealed novel functions of DNMT3A during gastric carcinogenesis, in which the de-regulation of the DNMT3A-p18^INK4C^ axis plays a critical role in cell cycle progression.

## Methods

### Tissue samples

A total of 35 pairs of GC tissue and adjacent non-tumor tissue specimens were collected between 2011 and 2013 at the Third Affiliated Hospital of Harbin Medical University. All patients were newly diagnosed and had not undergone any previous treatment. All tissue samples were obtained from GC patients during surgery and immediately snap frozen in liquid nitrogen until RNA and protein extraction. This study was reviewed and approved by the Committee for Ethical Review of Research at the Third Affiliated Hospital of Harbin Medical University in China, and the patients signed informed consent forms. The methods were carried out in accordance with the approved guidelines.

### Cell culture

GC cell lines (AGS and BGC-823) and a GES-1 immortalized normal human gastric cell line were obtained from the Cell Bank of the Chinese Academy of Science. The GC cell line MKN45 was kindly provided by assistant researcher Xiang-Ming Yan from the Shanghai Advanced Research Institute at the Chinese Academy of Science. All cell lines were maintained in RPMI-1640 medium supplemented with 10% fetal bovine serum (Invitrogen, Carlsbad, CA), 100 U/ml of penicillin and 100 mg/ml streptomycin (Invitrogen, Carlsbad, CA) in a humidified incubator with a 5% CO_2_ atmosphere at 37 °C. The GC cell line MKN45, which has lower DNMT3A expression, was chosen to perform a gain-of-function experiment. The GC cell line AGS, which has higher DNMT3A expression, was selected to perform a loss-of-function experiment. The GC cell line BGC-823, which has intermediate DNMT3A expression, was chosen to perform both gain- and loss-of-function experiments

### Transfection

A pIRESpuro/Myc-DNMT3A construct was kindly provided by Dr. Gang-Ning Liang of the Jane Anne Nhol Division of Hematology, Norris Cancer Center (Los Angeles, CA, USA). Expression plasmids were transfected into MKN45 and BGC-823 cells using Lipofectamine 2000 (Invitrogen, Carlsbad, CA, USA) according to the manufacturer’s instructions. Cells were subsequently selected with medium containing 0.4 μg/ml puromycin (Clontech, USA) for 30 days, and polyclonal cell lines were established. A p18^INK4C^ construct (Cat. No. B0138) was purchased from GeneCopoeia (Rockville, MD, USA). For siRNA transfection, siRNAs targeting p18^INK4C^, DNMT1 and DNMT3B mRNA were designed and synthesized by GenePharma (Shanghai, China). In addition, vector-based short hairpin RNAs (shRNAs) against DNMT3A N-terminal sequences and a control shRNA, the sequence of which did not match any known human gene, were transfected into AGS and BGC-823 cells using Lipofectamine 2000 according to the manufacturer’s instructions and selected with medium containing 400 μg/μl G418 (GIBCO, Gaithersburg, MD) for 30 days. Cells that stably harbored the targeting vector were monitored using green fluorescent protein expression.

### Western blot

Western blots were performed using anti-DNMT3A (Abcam, Cambridge, UK) and mouse anti-cyclinD1, anti-CDK4, anti-CDK6, anti-p18^INK4C^, and anti-CDKN1B which were purchased as part of a Cell Cycle Regulation Sampler Kit (Cell Signaling Technology). Mouse anti-*β-*actin was obtained from Sigma-Aldrich. Protein detection was performed with Super Signal Chemiluminescence Substrate (Pierce, USA).

### Cell growth and foci formation assay

A Cell Counting Kit-8 (CCK-8) (Dojindo Laboratories, Kumamoto, Japan) was used to measure cellular growth following the manufacturer’s instructions. Foci formation assay was performed by seeding 1 × 10^3^ cells in a 6-well plate. The surviving colonies (>50 cells per colony) were counted following crystal violet (Invitrogen, Carlsbad, CA, USA) staining. All experiments were independently repeated at least three times.

### Cell cycle analysis

Approximately 1 × 10^6^ cells were trypsinized, washed twice with phosphate-buffered saline containing 1% fetal bovine serum and incubated in phosphate-buffered saline containing 0.02% TritonX-100, 0.1 mg/ml RNase (Sigma-Aldrich) and 10 μg/ml propidium iodide (Sigma-Aldrich) for 30 min at 37 °C. Cell cycle distribution was examined by flow cytometry using a FACScan flow cytometer (Becton Dickinson & Co., San Jose, CA, USA). The relative number of cells in each phase of the cell cycle was analyzed using Modfit program (Verity Software House Inc., Topsham, ME, USA).

### Gene expression profiles and data analysis

Affymetrix GeneChip Human Transcriptome Array 2.0 was used for microarray analysis. Microarray hybridization was performed by the Shanghai Biotechnology Corporation (Shanghai, China) using standard Affymetrix procedures. Raw microarray data were acquired using GCOS1.2 software from Affymetrix. The raw data were preprocessed using robust multiarray analysis (RMA) with a log base 2 (log_2_) transformation. Gene ontology (GO) analysis was performed to identify the GO terms associated with the differentially expressed mRNA.

### Tumor xenograft mouse model

Approximately 1 × 10^7^ cells were injected subcutaneously into the right dorsal flanks of 4-week-old nude mice (n = 5 per group). Tumor formation was monitored over a 4-week period. Tumor volume was measured weekly and was calculated by the following formula: *V *= 0.5 × *L *× *W*^*2*^. All animal experiments were conducted in accordance with the institutional standard guidelines of Jiangning Hospital of Nanjing.

### Bisulfite sequencing

Genomic DNA was extracted from cells and tissue specimens using the phenol-chloroform method. Bisulfite treatment was performed using a CpGenome^TM^ Universal DNA Modification Kit (Millipore, USA), following the manufacturer’s instructions. Modified DNA was amplified, and PCR products were gel-purified and sub-cloned into a pMD19-T vector system (TAKARA, Japan). Ten colonies were sequenced to assess the degree of methylation at each CpG site. The PCR products of the tissue specimens were measured using Sequenom MassARRAY platform (Sequenom, Inc., San Diego, CA). The primers used are listed in Supplementary Table S1.

### Chromatin immunoprecipitation assay and ChIP-PCR

The ChIP experiments were performed using an EZ-Magna ChIP G kit (Upstate Biotechnology, Lake Placid, NY, USA) following the manufacturer’s instructions. The primers used to amplify the precipitated DNA fragments are listed in Supplementary Table S2.

### 5-Aza treatment

The DNMT3A overexpression cell line MKN45 was seeded at a density 1 × 10^6^ cells/ml in 10-cm dishes. After 16 h of culturing, the cells were treated with the indicated concentration of the DNA methylation inhibitor 5-Aza. Cell culture medium was replaced with freshly added 5-Aza for every 24 h and the total incubation time was 96 h. The cells were harvested for RNA extractions after treatment.

### Statistical analysis

The protein expression level of DNMT3A and the mRNA expression levels of p18^INK4C^, DNMT1 or DNMT3B in GC patients and the paired adjacent non-tumor tissues were compared using Wilcoxon’s test. The correlation between the DNMT3A and p18^INK4C^ expression levels was analyzed using Spearman’s test. The correlation between DNMT3A expression and pathological features was analyzed with the Pearson *χ*^*2*^ test. Independent Student’s *t*-test was used to compare the results, which are expressed as the mean ± SD between any two preselected groups. A *P*-value of less than 0.05 was considered statistically significant.

## Additional Information

**How to cite this article**: Cui, H. *et al.* DNA methyltransferase 3A promotes cell proliferation by silencing CDK inhibitor p18^INK4C^ in gastric carcinogenesis. *Sci. Rep.* **5**, 13781; doi: 10.1038/srep13781 (2015).

## Supplementary Material

Supplementary Information

## Figures and Tables

**Table 1:** Clinicopathological correlation of DNMT3A expression in GC cases.

**Feature**	**N ≥ T**^a^	**N < T**	***P*****-value**
Gender (n = 35)			
Female	9	16	
Male	4	6	0.825
Age (n = 35)			
≤60 years	7	15	
>60 years	6	7	0.396
Lauren’s classification (n = 35)			
Diffuse type	7	12	
Intestinal type	6	10	0.968
Histologic grade (n = 33)			
Poor	4	18	
Moderate	7	4	
High^b^	0	0	**0.009**^c^
TNM staging (n = 35)			
Stage I/II	2	5	
Stage III/IV	11	17	0.600
Vascular invasion (n = 35)			
Yes	2	7	
No	11	15	0.282
Lymph node metastasis (n = 35)			
Yes	9	19	
No	4	3	0.221

^a^N: non-tumor tissues; T: tumor tissues.

^b^No cases in this pathological classification.

^c^Significant differences are shown in bold.
