# Supplementary material for: DNA methyltransferase 3A promotes cell proliferation by silencing CDK inhibitor p18INK4C in gastric carcinogenesis
Source: Sci Rep. 2015 Sep 9;5:13781. doi: 10.1038/srep13781 (PMC4563369; doi:10.1038/srep13781)

Supplementary Information

DNA methyltransferase 3A promotes cell proliferation by silencing CDK inhibitor p18^{INK4C} in gastric carcinogenesis

He Cui[1], Chengcheng Zhao[1], Pihai Gong[1], Ling Wang[1], Huazhang Wu[1], Kun Zhang[2], Rongping Zhou[3], Li Wang[4], Ting Zhang[4], Sheng Zhong[4,*] & Hong Fan[1,*]

Table S1 Primers used in methylation analysis of INK4 members

Gene	Sequences	
	sense	antisense
**For BGS**		
P15^{INK4B}	GGTTGGTTTTTTATTTTGTTAGAG	ACCTAAACTCAACTTCATTACCCTC
P16^{INK4A}	AAAAATGGGTTAGATATAAAGGATT	CCTCTTCTAAATTTAAAAAACAAAC
P18^{INK4C}	TAGGAATTGGGGTAGTTGGGG	TTACCTCTCAAAAAAAATACCARTTT
P19^{INK4D}	TAATGAATGTTTATTAGTAGTGG	TCCTAATTAATCAAAATATAACAACC

Table S2 Primers used in ChIP analysis of P18^{INK4C} promoter

Fragments	Sequences	
	sense	antisense
F1	TGTTGCTAATACTCCCAG	TTAGAAGTGGCTTGGATT
F2	ATTGGTGTCGGATGATTA	GACTGCCACTAAGCACAG
F3	CATAAAATTCAGTCTACACA	TAAGACGGTTGTGGAGGGC
F4	GAACGACTCCCTTTATGC	TCTCCACCTCCTCCCGTCAA
F5	TCACAGACTCAAGCCCGCCA	TTAAGGAGGCTCGGCAGA
F6	GAGCGTGCGAGACTGCGAGC	CTGCTTCTGTTGCCTCTC

Supplementary Figure legends

Figure S1 Generation of stable cell lines with DNMT3A RNAi or DNMT3A overexpression. (a) Western blot was performed to detect the efficiency of RNAi with DNMT3A siRNA in AGS and BGC-823 cells. **(b)** Western blot was performed to detect the efficiency of DNMT3A overexpression in MKN45 and BGC-823 cells. *β*-actin was used as a loading control.

Figure S2 DNMT3A promotes cell growth *in vitro* and *in vivo*. (a) The growth rates of MKN45-DNMT3A cells were detected by CCK-8 proliferation assay and are shown as the mean ± SD of three independent experiments (*P<0.05). **(b)** Images of tumors formed in the nude mice injected with the indicated cells.

Figure S3 The effect of DNMT3A on the expression of CDKN1B. (a) Western blot analysis of CDKN1B expression in DNMT3A knockdown cells AGS or BGC-823. *β*-actin was used as a loading control (left panels). The band intensities were quantified and normalized to *β*-actin intensities with *ImageJ* software (right panels).

Figure S4 The effect of DNMT3B or DNMT1 on the expression of INK4 members. (a and b) Relative mRNA expression of INK4 members was detected in DNMT3B or DNMT1 knockdown AGS cells by qPCR. *β*-actin was

used as an internal control. **(c and d)** Scatter plots of relative DNMT1 or DNMT3B mRNA expression in 35 GC and adjacent non-tumor tissues. In both panels, the red lines indicate the mean ± SEM. **(e)** The relative mRNA expression of DNMT3B or DNMT1 in AGS, BGC-823 and MKN45 GC cells used in this study. GES-1, the immortalized normal human gastric cell line, was used as a control.

Cui et al; Supplementary Figure 1

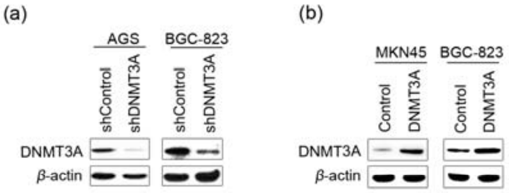

Cui et al; Supplementary Figure 2

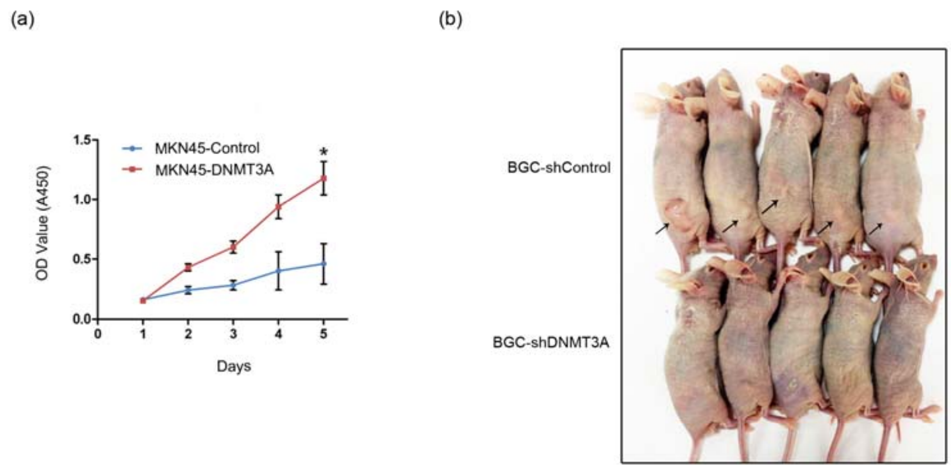

Cui et al; Supplementary Figure 3

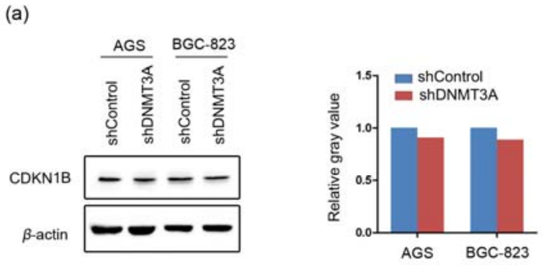

Cui et al; Supplementary Figure 4

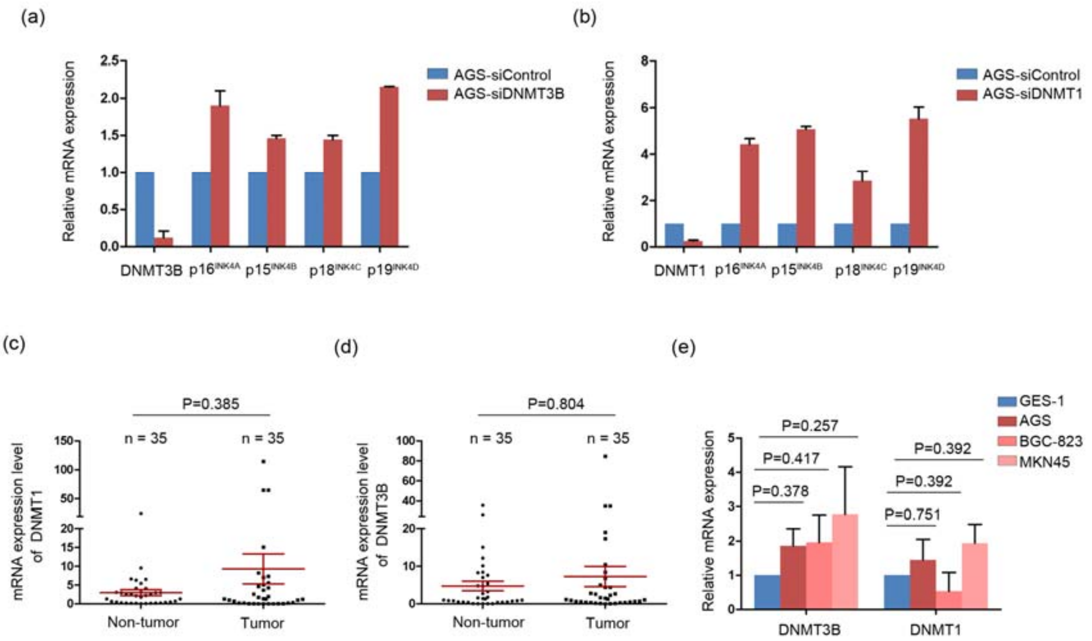

Supplement: Supplementary Information [file srep13781-s1.pdf]
